# Adult Pleomorphic Rhabdomyosarcomas: Assessing Outcomes Associated with Radiotherapy and Chemotherapy Use in the National Cancer Database

**DOI:** 10.1155/2021/9712070

**Published:** 2021-03-13

**Authors:** Vishruth K. Reddy, Varsha Jain, Robert J. Wilson II, Lee P. Hartner, Mark Diamond, Ronnie A. Sebro, Kristy L. Weber, Robert G. Maki, Jacob E. Shabason

**Affiliations:** ^1^Department of Radiation Oncology, University of Pennsylvania, Philadelphia, PA, USA; ^2^Department of Orthopedic Surgery, University of Pennsylvania, Philadelphia, PA, USA; ^3^Division of Hematology/Oncology, Department of Medicine, University of Pennsylvania, Philadelphia, PA, USA; ^4^Department of Radiology, University of Pennsylvania, Philadelphia, PA, USA; ^5^Department of Genetics, University of Pennsylvania, Philadelphia, PA, USA; ^6^Department of Biostatistics, Epidemiology and Bioinformatics, University of Pennsylvania, Philadelphia, PA, USA

## Abstract

**Purpose:**

Practice patterns for treatment of localized adult pleomorphic rhabdomyosarcoma (PRMS) remain quite variable given its rarity. Current national guidelines recommend management similar to that of other high-grade soft tissue sarcomas (STS), which include surgery with perioperative radiation (RT) with or without chemotherapy. Using the National Cancer Database (NCDB), we assessed practice patterns and overall outcomes of patients with localized PRMS. *Patients and Methods*. Patients with stage II/III PRMS treated with surgical resection from 2004 to 2015 were identified from the NCDB. Predictors of RT and chemotherapy use were assessed using multivariable logistic regression analysis. The association of radiation and chemotherapy status on overall survival was assessed using Kaplan–Meier and Cox proportional hazards analyses.

**Results:**

Of 243 total patients, RT and chemotherapy were not uniformly utilized, with 44% receiving chemotherapy and in those who did not undergo amputation 62% receiving RT. In those who did not undergo amputation, RT was associated with improved survival on both univariate (HR: 0.49, 95% CI 0.32–0.73, *P* < 0.001) and multivariate analysis (HR: 0.40, 95% CI 0.26–0.62, *P* < 0.001), corresponding to greater 5-year overall survival (59% vs. 38%, *P* < 0.001). Chemotherapy was associated with a higher rate of 5-year overall survival (63% vs. 39%, *P* < 0.001). However, the survival benefit of chemotherapy did not reach statistical significance on multivariate analysis (HR: 0.65, 95% CI 0.41–1.03, *P*=0.064). Notable predictors of omission of RT included female gender (OR: 0.40, 95% CI 0.22–0.74, *P* < 0.01) and age ≥ 70 (OR: 0.55, 95% CI 0.30–1.00, *P*=0.05). Correspondingly, factors associated with omission of chemotherapy included age ≥70 (OR: 0.17, 95% CI 0.08–0.39, *P* < 0.001).

**Conclusions:**

A significant proportion of patients with localized adult PRMS are not receiving RT. Likewise, use of chemotherapy was heterogeneous. Our findings note potential benefits and underutilization of RT, for which further investigation is warranted.

## 1. Introduction

Soft tissue sarcomas (STS) are mesenchymal malignancies that comprise a small proportion (<1%) of all cancers diagnosed yearly in the United States [1]. Adult pleomorphic rhabdomyosarcomas (PRMS) are a rare subset of STS for which the optimal management is not well-defined [2]. Given their rarity, limited data exists as to their optimal management, though it is often best achieved with multidisciplinary care involving surgery, radiation oncology, medical oncology, radiology, and pathology [2]. National guidelines recommend treatment of adult PRMS similarly to other high-grade STS, with the addition of radiotherapy (RT) to surgery, largely relying on randomized data demonstrating improvement in local control with the addition of RT for high-grade STS [2–5]. Chemotherapy is sometimes given for high-grade disease, though its role remains controversial [6, 7]. Just as with other high-grade STS, there appears to exist heterogeneity in RT and chemotherapy use amongst providers [8–10]. The aim of this study was to assess overall outcomes for patients with localized adult PRMS, identify which patients receive RT and chemotherapy, and evaluate the association between RT and chemotherapy use and survival in patients diagnosed with localized PRMS using the National Cancer Database (NCDB).

## 2. Methods

### 2.1. Data Source

The study population was identified from the National Cancer Database (NCDB), a national cancer registry jointly sponsored by the American College of Surgeons and the American Cancer Society that draws upon hospital registry data from more than 1,500 Commission on Cancer- (CoC-) accredited facilities in the United States [11, 12]. The dataset captures more than 70% of incident cancers and comprises more than 34 million unique cancer cases [11, 12]. Data are collected prospectively from Commission on Cancer-accredited program cancer registries with nationally standardized data-coding definitions.

### 2.2. Study Population

Inclusion criteria for the cohort consisted of patients with non-metastatic PRMS from 2004 to 2015 who were treated with surgical resection. Patients with PRMS arising in the head, neck, extremities, thorax, trunk, abdomen, and pelvis were included. Only those patients who did not undergo amputation were included in the assessment of outcomes associated with receipt of RT, as RT would not be indicated after an amputation.

### 2.3. Patient Cohorts and Variables

The covariates examined included sex, age, race, population density of patient residence (classified as metropolitan, urban, or rural), facility geographic location, facility type (nonacademic or academic), distance to treatment facility, educational attainment (defined as percentage of population in patient's ZIP code without a high school degree), income (defined as median income in patient's ZIP code), Charlson/Deyo comorbidity score [13], primary site of tumor, tumor size, tumor grade, receipt of chemotherapy and RT, and year of treatment.

### 2.4. Statistical Analysis

The independent effect of receipt of RT or chemotherapy on hazard of death in patients with localized PRMS disease was assessed using Cox proportional hazards analyses. All covariates achieving a threshold significance of *P* < 0.1 on univariate analysis were included in the multivariable model. The Kaplan-Meier estimator and log-rank test were used to compare OS between the cohorts. To more robustly account for baseline difference between cohorts, a secondary survival analysis was performed using propensity score (PS) matched cohorts for those treated with RT. Those treated with RT were matched to those in whom RT was omitted. This was done using 1-to-1 nearest neighbor matching without replacement [14] (matched for all covariates listed in Table 1). Absolute standardized differences of <0.1 between baseline covariates following matching was accepted as a measure of adequate balance [15]. A Cox survival analysis was then repeated on the matched cohorts to estimate the hazard of death associated with receipt of RT. A two-tailed *P* value < 0.05 was considered statistically significant. In addition, a multivariable logistic regression model was constructed using all baseline covariates to assess the independent effect of each covariate on the odds of being treated with RT and chemotherapy. Statistical analyses were performed using Stata SE, version 15.0 (StataCorp, College Station, TX).

## 3. Results

### 3.1. Baseline Clinical Characteristics

**Figure 1:**
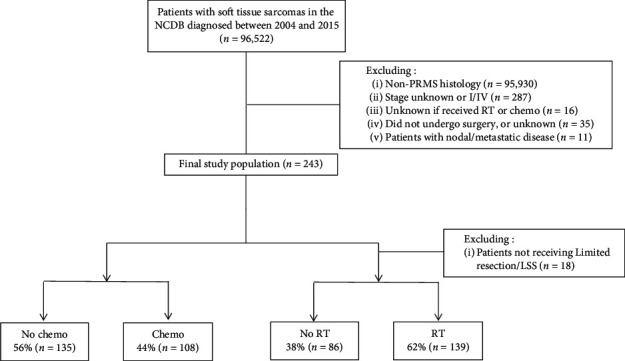
Consolidated Standards of Reporting Trials (CONSORT) diagram of the patient cohort; NCDB: National Cancer Database.

A total of 243 patients met study inclusion criteria (Figure 1). Complete patient characteristics are shown in Table 2. Notably, the median age of the patient cohort was 64 years (range, 22–90 years). The majority of patients were men (62%), non-Hispanic White (79%), and without significant comorbid illness (81%). In terms of disease characteristics, most patients had tumors arising from the extremity (66%), grade III disease (95%), and tumor size >5 cm (79%). Overall, RT and chemotherapy were not uniformly utilized in the management of these patients with 44% receiving chemotherapy and in those who did not undergo amputation only 62% receiving RT. The majority of patients who received chemotherapy with modality specified received multi-agent therapy (91%). Of those who received RT, the majority received RT adjuvantly (68%) rather than neoadjuvantly (32%).

### 3.2. Impact of Radiotherapy and Chemotherapy on Overall Survival

**Figure 2:**
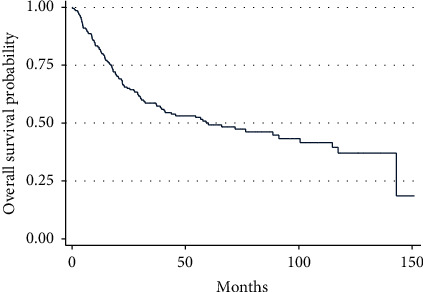
Overall survival in patients with localized PRMS.

**Figure 3:**
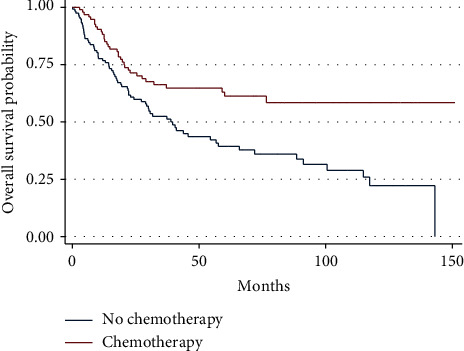
Overall survival as a function of receipt of chemotherapy in patients with localized PRMS (log rank *P* < 0.001).

The median survival for all patients with localized PRMS was 60.1 months, with a 5-year overall survival of 50% (95% CI 42.4–57.2) (Figure 2). When analyzing the entire population of patients with stage II/III disease, the use of chemotherapy was associated with a decreased hazard of death on univariate analysis (HR: 0.50, 95% CI 0.33–0.75, *P* < 0.001) (Table 3). The 5-year overall survival was 63% for those who received chemotherapy vs. 39% for those who did not (*P* < 0.001) (Figure 3). However, the benefit of chemotherapy was not retained on multivariate analysis (HR: 0.65, 95% CI 0.41–1.03, *P*=0.064) (Table 3).

**Figure 4:**
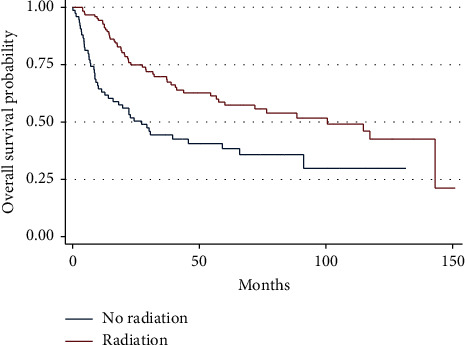
Overall survival as a function of receipt of radiotherapy status in patients with localized PRMS who did not undergo amputation (log rank *P* < 0.001).

Analysis of the subset of patients not treated with amputation, as there would not be an indication for RT following amputation, noted that patients treated with RT had an improved 5-year OS (59% vs. 38%, *P* < 0.001) (Figure 4). Correspondingly, RT was associated with a decreased hazard of death on both univariate (HR: 0.49, 95% CI 0.32–0.73, *P* < 0.001) and multivariate analysis (HR: 0.40, 95% CI 0.26–0.62, *P* < 0.001) (Table 1). The improvement in OS remained after MV-PS analysis (HR: 0.49, 95% CI 0.27–0.90, *P* < 0.05) (Table 1).

### 3.3. Factors Associated with Receipt of Chemotherapy and Radiotherapy

On multivariable analysis, notable predictors of omission of chemotherapy included older age (≥70 years) (OR: 0.17, 95% CI 0.08–0.39, *P* < 0.001) (Table 4). Correspondingly, on multivariate analysis, factors associated with the omission of RT in the population that did not undergo amputation included female gender (OR: 0.40, 95% CI 0.22–0.74, *P* < 0.01) and older age (≥70 years) (OR: 0.55, 95% CI 0.30–1.00, *P*=0.05) (Table 5).

## 4. Discussion

We utilized a national cancer registry to evaluate the management of patients with localized adult PRMS. To our knowledge, this is the most comprehensive study to examine patterns of care and the association between RT and chemotherapy use and survival in a real-world cohort of patients. National guidelines recommend that treatment for adult PRMS corresponds to that of other high-grade STS, which would include the addition of RT and consideration of systemic therapy in addition to surgical resection [2]. Indeed, randomized data has demonstrated improvement in local control with the addition of RT for patients with high-grade STS [3–5]. The benefit of adjuvant chemotherapy is more controversial, as many trials over the past few decades have noted disparate results [16–23]. A meta-analysis demonstrated a benefit in overall recurrences and survival with chemotherapy [6], while a more recent study showed no survival benefit [7].

In regard to overall outcomes for patients with localized PRMS, prior studies are limited [24–26]. Our study notes that the overall median survival for this cohort is 60.1 months. Perhaps the most significant finding of our study was that, in patients with localized PRMS, RT was associated with longer survival yet potentially underutilized, with only 62% of these patients receiving RT over the study period (2004–2015), for which further investigation is warranted. In this group, there was a higher rate of overall survival with decreased hazard of death on multivariate analysis (HR: 0.40, 95% CI 0.26–0.62, *P* < 0.001). Although chemotherapy is associated with improved survival in patients with localized PRMS on univariate analysis, the observed benefit was not retained on multivariate analysis.

Other notable findings from our study were that women and older populations were less likely to receive RT, suggesting that these populations may be additionally vulnerable to omission of RT for adult PRMS. Our study is consistent with several others which have identified undertreatment of females in comparison to their male counterparts for other disease sites and modalities of cancer care, which may be due to a number of unmeasured factors ranging from implicit physician biases to differences in patient treatment goals [27–32]. Moreover, we have previously shown that older populations are less likely to receive perioperative RT for STS [10], likely due to a number of potential factors that others have investigated, including physician-based factors such as hesitancy to recommend more intensive treatment due to preconceived biases in regard to their frailty, as well as patient-related factors such as prioritization of immediate convenience and quality of life over long-term outcomes and survival [33–35]. These same factors may also be contributing to chemotherapy omission in elderly patients with PRMS, as noted in our analysis.

Interestingly, we noted that the majority of patients who received radiotherapy received it adjuvantly. Studies of practice patterns in the management of other soft tissue sarcomas have noted that radiotherapy has been predominantly utilized adjuvantly [36], potentially in part due to surgeon preference, though with the proportion of those receiving neoadjuvant treatment increasing over time. Indeed, recent studies have demonstrated that neoadjuvant treatment may offer select benefits for patients with extremity STS treated with RT, including smaller treatment volume and lower dose, which translates to a lower risk of late radiation-induced complications, such as edema, fibrosis, and joint stiffness [37]. However, neoadjuvant RT is associated with a higher risk of acute wound complications compared to adjuvant RT [37].

The strengths of the present study include a modern cohort of patients treated for PRMS and adjustment for a range of patient- and facility-level variables. Our study has several notable limitations given its retrospective design and reliance on the content and accuracy of information included in the NCDB. Additionally, there is inherent selection bias associated with the retrospective nature of this analysis. Despite these limitations, however, we aimed to more robustly account for baseline difference between cohorts with propensity score matching, with our results demonstrating that the survival benefit associated with receipt of radiotherapy remained. It is also possible that we were unable to account for several unmeasured confounders such as patient preferences, physician attitudes, referral patterns, and quality of care received, which impacted patient selection and management. These factors amongst others may have confounded our analyses and may in part explain why there was an associated survival benefit with chemotherapy on univariate but not multivariate analysis. Another limitation of our study is that our dataset did not allow for assessment of local recurrence-free survival. Indeed, while we would speculate that the improved survival associated with radiotherapy may be at least in part due to inhibition of local progression, we were unable to specifically evaluate this. Additionally, the difficulty in ensuring accuracy of pathological diagnosis with adult PRMS remains an ongoing challenge for providers who manage this disease as well as studies of patient outcomes. Finally, it is important to keep in mind that this study included PRMS of various sites of origin, which certainly impacts both resectability and overall clinical outcomes.

In conclusion, we demonstrate that a sizeable proportion of patients with localized adult PRMS are not receiving RT and chemotherapy, likely due to limited data in regard to the management of these patients. Additionally, our analysis also reflects that certain subgroups may be particularly vulnerable to omission of treatment with potential to adversely impact outcomes. Our study notes potential benefits of RT in particular, for which further investigation is warranted.

## Figures and Tables

**Table 1:** Factors associated with overall survival in patients with localized disease who did not undergo amputation.

	Univariate		Multivariate		Propensity score matched	
	HR (95% CI)	*P* value	HR (95% CI)	*P* value	HR (95% CI)	*P* value
Receipt of radiation						
No	1		1		1	
Yes	0.49 (0.32–0.73)	<0.001	0.40 (0.26–0.62)	<0.001	0.49 (0.27–0.90)	<0.05
Receipt of chemotherapy						
No	1		1		—	—
Yes	0.51 (0.33–0.78)	0.002	0.70 (0.42–1.16)	0.170	—	—
Age						
<70 years	1		1		—	—
≥70 years	2.55 (1.71–3.82)	<0.001	1.40 (0.70–2.78)	0.343	—	—
Gender						
Male	1		—	—	—	—
Female	1.08 (0.71–1.62)	0.723	—	—	—	—
Race						
Non-Hispanic White	1		—	—	—	—
Non-Hispanic Black	0.77 (0.39–1.53)	0.456	—	—	—	—
Hispanic	0.35 (0.11–1.10)	0.073	—	—	—	—
Other	0.77 (0.31–1.90)	0.567	—	—	—	—
Facility area						
Metropolitan	1		1		—	—
Urban	0.35 (0.14–0.86)	0.022	0.31 (0.12–0.80)	0.016	—	—
Rural	1.55 (0.49–4.90)	0.458	1.75 (0.51–6.01)	0.373	—	—
Unknown	1.72 (0.63–4.71)	0.290	1.82 (0.61–5.39)	0.281	—	—
Facility location						
East	1		1		—	—
South	0.99 (0.56–1.76)	0.980	0.87 (0.46–1.63)	0.659	—	—
Central	1.17 (0.64–2.13)	0.608	1.19 (0.62–2.31)	0.603	—	—
West	0.83 (0.43–1.60)	0.576	0.91 (0.46–1.82)	0.788	—	—
Unknown	0.35 (0.13–0.94)	0.036	.	.	—	—
Facility type						
Non-academic	1		1		—	—
Academic	0.99 (0.66–1.49)	0.961	1.04 (0.66–1.66)	0.854	—	—
Unknown	0.34 (0.14–0.87)	0.025	0.66 (0.22–1.95)	0.447	—	—
Insurance						
Commercial	1		1		—	—
Medicare	2.30 (1.50–3.52)	<0.001	1.43 (0.70–2.90)	0.322	—	—
Medicaid	1.17 (0.46–2.98)	0.743	1.26 (0.45–3.52)	0.664	—	—-
Uninsured	.	.	.	.	—	—
Other	0.77 (0.10–5.59)	0.792	1.17 (0.15–9.33)	0.884	—	—
Distance to treatment facility						
≤40 miles	1		—	—	—	—
>40 miles	0.94 (0.60–1.47)	0.783	—	—	—	—
Zip code education level						
≥21%	1		1		—	—
13%–20.9%	2.60 (1.26–5.34)	0.009	3.17 (1.48–6.76)	0.003	—	—
7%–12.9%	1.75 (0.85–3.59)	0.131	1.90 (0.89–4.05)	0.098	—	—
<7%	1.96 (0.96–4.01)	0.066	2.02 (0.94–4.35)	0.071	—	—
Zip code income level						
<38,000	1		—	—	—	—
38,000–47,999	1.09 (0.57–2.08)	0.795	—	—	—	—
48,000–62,999	0.90 (0.48–1.66)	0.725	—	—	—	—
≥63,000	1.07 (0.59–1.96)	0.818	—	—	—	—
Charlson/Deyo score						
0	1		—	—	—	—
1	1.76 (1.02–3.04)	0.043	—	—	—	—
2	1.61 (0.51–5.12)	0.420	—	—	—	—
3	2.48 (0.78–7.91)	0.125	—	—	—	—
Primary site						
Head and neck	1		—	—	—	—
Upper extremity	1.24 (0.40–3.85)	0.709	—	—	—	—
Lower extremity	1.74 (0.63–4.84)	0.287	—	—	—	—
Thorax	1.96 (0.62–6.14)	0.251	—	—	—	—
Abdomen/pelvis	2.34 (0.80–6.79)	0.119	—	—	—	—
Other/NOS	.	.	—	—	—	—
Tumor size						
<5 cm	1		1		—	—
5.1–10 cm	1.56 (0.87–2.79)	0.137	1.45 (0.78–2.71)	0.242	—	—
10.1–15 cm	1.87 (0.97–3.62)	0.062	1.55 (0.74–3.25)	0.241	—	—
>15 cm	3.82 (1.99–7.32)	<0.001	4.06 (1.96–8.40)	<0.001	—	—
Grade						
II	1		1		—	—
III	2.85 (0.70–11.57)	0.143	2.07 (0.48–8.92)	0.327	—	—
Year of diagnosis						
2004–2007	1		—	—	—	—
2008–2011	0.69 (0.44–1.11)	0.124	—	—	—	—
2012–2015	0.68 (0.39–1.18)	0.172	—	—	—	—

**Table 2:** Baseline patient characteristics.

	Total	%
Total, n	243	100
Surgery type		
Resection or LSS^∗^	225	*93*
Amputation	18	*7*
Receipt of radiotherapy^*ϕ*^		
No	102	*42*
Yes	141	*58*
Receipt of chemotherapy		
No	135	*56*
Yes	108	*44*
Age		
<70 years	158	*65*
≥70 years	85	*35*
Gender		
Male	151	*62*
Female	92	*38*
Race		
Non-Hispanic White	191	*79*
Non-Hispanic Black	20	*8*
Hispanic	18	*7*
Other	14	*6*
Facility area		
Metropolitan	202	*83*
Urban	26	*11*
Rural	8	*3*
Unknown	7	*3*
Insurance		
Commercial	113	*47*
Medicare	102	*42*
Medicaid	17	*7*
Uninsured	3	*1*
Other	8	*3*
Zip code education level		
≥21%	40	*16*
13%–20.9%	59	*24*
7%–12.9%	77	*32*
<7%	67	*28*
Zip code income level		
<38,000	41	*17*
38,000–47,999	53	*22*
48,000–62,999	74	*30*
≥63,000	75	*31*
Facility type		
Non-academic	103	*42*
Academic	114	*47*
Unknown	26	*11*
Facility location		
East	45	*19*
South	68	*28*
Central	53	*22*
West	51	*21*
Unknown	26	*11*
Distance to treatment facility		
≤40 miles	171	*70*
>40 miles	72	*30*
Charlson/Deyo score		
0	196	*81*
1	36	*15*
2	7	*3*
3	4	*2*
Primary site		
Head and neck	12	*5*
Upper extremity	40	*16*
Lower extremity	120	*49*
Thorax	23	*9*
Abdomen/pelvis	46	*19*
Other/NOS	2	*1*
Grade		
II	11	*5*
III	232	*95*
Tumor size		
<5 cm	51	*21*
5.1–10 cm	102	*42*
10.1–15 cm	52	*21*
>15 cm	38	*16*
Clinical stage		
II	51	*21*
III	192	*79*
Year of diagnosis		
2004–2007	74	*30*
2008–2011	78	*32*
2012–2015	91	*37*

^∗^ *Limb-sparing surgery*. ^*ϕ*^When considering only those patients who did not undergo amputation, for whom RT would not be indicated, 86 (38%) did not receive radiotherapy and 139 (62%) received radiotherapy.

**Table 3:** Factors associated with overall survival in patients with localized disease.

	Univariate analysis		Multivariate analysis	
	HR (95% CI)	*P* value	HR (95% CI)	*P* value
Receipt of radiation				
No	1		1	
Yes	0.50 (0.34–0.74)	<0.001	0.48 (0.32–0.72)	<0.001
Receipt of chemotherapy				
No	1		1	
Yes	0.50 (0.33–0.75)	0.001	0.65 (0.41–1.03)	0.064
Age				
<70 years	1		1	
≥70 years	2.50 (1.70–3.67)	<0.001	1.55 (0.83–2.90)	0.171
Gender				
Male	1		—	—
Female	1.11 (0.75–1.64)	0.594	—	—
Race				
Non-Hispanic White	1		—	—
Non-Hispanic Black	0.72 (0.36–1.43)	0.345	—	—
Hispanic	0.43 (0.16–1.18)	0.101	—	—
Other	0.75 (0.30–1.85)	0.530	—	—
Facility area				
Metropolitan	1		1	
Urban	0.48 (0.22–1.04)	0.062	0.48 (0.21–1.09)	0.078
Rural	1.54 (0.49–4.88)	0.461	1.81 (0.53–6.20)	0.344
Unknown	1.70 (0.62–4.63)	0.302	2.73 (0.93–8.00)	0.066
Facility location				
East	1		1	
South	1.16 (0.67–2.02)	0.601	1.09 (0.60–1.98)	0.781
Central	1.27 (0.71–2.29)	0.420	1.52 (0.77–2.99)	0.228
West	1.01 (0.54–1.88)	0.971	1.14 (0.59–2.18)	0.701
Unknown	0.37 (0.14–1.00)	0.049	0.85 (0.28–2.56)	0.777
Facility type				
Non-academic	1		1	
Academic	0.95 (0.64–1.41)	0.803	0.93 (0.61–1.43)	0.753
Unknown	0.32 (0.13–0.82)	0.017	.	.
Insurance				
Commercial	1		1	
Medicare	2.34 (1.55–3.53)	<0.001	1.38 (0.72–2.62)	0.332
Medicaid	1.21 (0.51–2.88)	0.660	0.79 (0.31–2.01)	0.626
Uninsured	.	.	.	.
Other	0.71 (0.10–5.18)	0.736	0.82 (0.11–6.26)	0.845
Distance to treatment facility				
≤40 miles	1		—	—
>40 miles	1.04 (0.68–1.59)	0.859	—	—
Zip code education level				
≥21%	1		—	—
13%–20.9%	1.97 (1.04–3.71)	0.037	—	—
7%–12.9%	1.28 (0.68–2.43)	0.445	—	—
<7%	1.51 (0.80–2.83)	0.202	—	—
Zip code income level				
<38,000	1		—	—
38,000–47,999	0.91 (0.50–1.65)	0.747	—	—
48,000–62,999	0.78 (0.44–1.39)	0.402	—	—
≥63,000	0.88 (0.50–1.53)	0.646	—	—
Charlson/Deyo score				
0	1		1	
1	1.90 (1.14–3.15)	0.013	1.71 (0.98–2.99)	0.059
2	1.63 (0.51–5.19)	0.406	1.35 (0.41–4.48)	0.624
3	2.45 (0.77–7.79)	0.130	0.95 (0.27–3.33)	0.939
Primary site				
Head and neck	1		—	—
Upper extremity	1.25 (0.41–3.81)	0.689	—	—
Lower extremity	1.80 (0.65–4.98)	0.259	—	—
Thorax	2.07 (0.67–6.41)	0.209	—	—
Abdomen/pelvis	2.41 (0.83–6.97)	0.105	—	—
Other/NOS	.	.	.	.
Tumor size				
<5 cm	1		1	
5.1–10 cm	1.50 (0.85–2.64)	0.162	1.40 (0.77–2.57)	0.271
10.1–15 cm	1.83 (0.97–3.45)	0.063	1.70 (0.84–3.45)	0.141
>15 cm	3.62 (1.94–6.74)	<0.001	3.23 (1.59–6.53)	0.001
Grade				
II	1		1	
III	2.93 (0.72–11.90)	0.132	2.09 (0.49–8.87)	0.320
Year of diagnosis				
2004–2007	1		—	—
2008–2011	0.70 (0.45–1.10)	0.121	—	—
2012–2015	0.71 (0.42–1.22)	0.215	—	—

**Table 4:** Factors associated with receipt of chemotherapy.

Receipt of chemotherapy	Univariate analysis		Multivariate analysis	
	OR (95% CI)	*P* value	OR (95% CI)	*P* value
Age				
<70 years	1		1	
≥70 years	0.15 (0.08–0.28)	<0.001	0.17 (0.08–0.39)	<0.001
Gender				
Male	1		—	—
Female	0.76 (0.45–1.28)	0.301	—	—
Race				
Non-Hispanic White	1		—	—
Non-Hispanic Black	2.42 (0.92–6.33)	0.072	—	—
Hispanic	0.50 (0.17–1.46)	0.205	—	—
Other	1.30 (0.44–3.85)	0.635	—	—
Facility area				
Metropolitan	1		—	—
Urban	1.05 (0.46–2.37)	0.915	—	—
Rural	0.17 (0.02–1.44)	0.105	—	—
Unknown	1.63 (0.35–7.45)	0.531	—	—
Facility location				
East	1		1	
South	0.70 (0.32–1.52)	0.367	0.88 (0.35–2.18)	0.775
Central	1.05 (0.47–2.34)	0.907	1.03 (0.41–2.59)	0.944
West	1.04 (0.46–2.33)	0.928	1.13 (0.45–2.86)	0.795
Unknown	5.75 (1.84–17.98)	0.003	3.65 (0.95–13.95)	0.059
Facility type				
Non-academic	1		1	
Academic	1.39 (0.81–2.41)	0.234	1.23 (0.65–2.34)	0.520
Insurance				
Commercial	1		1	
Medicare	0.29 (0.16–0.51)	<0.001	0.91 (0.42–1.96)	0.801
Medicaid	2.49 (0.76–8.10)	0.130	2.72 (0.72–10.24)	0.140
Uninsured	0.38 (0.03–4.34)	0.438	0.43 (0.03–5.48)	0.517
Other	0.26 (0.05–1.32)	0.103	0.33 (0.05–2.03)	0.233
Distance to treatment facility				
≤40 miles	1		—	—
>40 miles	.	.	—	—
Zip code education level				
≥21%	1		1	
13%–20.9%	0.95 (0.41–2.22)	0.910	1.03 (0.37–2.82)	0.961
7%–12.9%	1.81 (0.82–3.98)	0.140	1.64 (0.65–4.17)	0.298
<7%	2.16 (0.96–4.84)	0.062	2.72 (1.02–7.25)	0.045
Zip code income level				
<38,000	1		—	—
38,000–47,999	1.27 (0.54–2.96)	0.586	—	—
48,000–62,999	1.73 (0.79–3.82)	0.174	—	—
≥63,000	1.98 (0.90–4.36)	0.089	—	—
Charlson/Deyo score				
0	1		—	—
1	0.72 (0.35–1.49)	0.374	—	—
2	0.45 (0.09–2.39)	0.350	—	—
3	.	.	—	—
Primary site				
Extremity	1		—	—
Head and neck	4.27 (1.11–16.38)	0.034	—	—
Thorax	1.55 (0.65–3.73)	0.325	—	—
Abdomen/pelvis	1.00 (0.51–1.95)	0.995	—	—
Tumor size				
<5 cm	1		—	—
5.1–10 cm	1.28 (0.64–2.55)	0.486	—	—
10.1–15 cm	2.30 (1.04–5.06)	0.039	—	—
>15 cm	1.10 (0.46–2.60)	0.831	—	—
Grade				
II	1		—	—
III	0.65 (0.19–2.20)	0.493	—	—
Receipt of radiotherapy				
No	1		—	—
Yes	1.35 (0.80–2.26)	0.257	—	—
Year of diagnosis				
2004–2007	1		1	
2008–2011	2.12 (1.11–4.04)	0.023	1.75 (0.81–3.77)	0.152
2012–2015	0.93 (0.49–1.74)	0.810	0.84 (0.40–1.73)	0.633

**Table 5:** Factors associated with receipt of radiotherapy.

Receipt of radiotherapy	Univariate analysis		Multivariate analysis	
	OR (95% CI)	*P* value	OR (95% CI)	*P* value
Age				
<70 years	1		1	
≥70 years	0.62 (0.36–1.09)	0.096	0.55 (0.30–1.00)	0.052
Gender				
Male	1		1	
Female	0.50 (0.29–0.87)	0.014	0.40 (0.22–0.74)	0.003
Race				
Non-Hispanic White	1		—	—
Non-Hispanic Black	1.45 (0.53–4.00)	0.471	—	—
Hispanic	0.77 (0.27–2.21)	0.621	—	—
Other	4.02 (0.87–18.50)	0.074	—	—
Facility area				
Metropolitan	1		—	—
Urban	1.31 (0.53–3.21)	0.556	—	—
Rural	1.64 (0.31–8.66)	0.562	—	—
Unknown	1.64 (0.31–8.66)	0.562	—	—
Facility location				
East	1		—	—
South	0.61 (0.27–1.38)	0.235	—	—
Central	0.96 (0.40–2.28)	0.922	—	—
West	0.90 (0.37–2.15)	0.808	—	—
Unknown	1.53 (0.49–4.75)	0.466	—	—
Facility type				
Non-academic	1		—	—
Academic	1.14 (0.65–2.01)	0.648	—	—
Insurance				
Commercial	1		—	—
Medicare	0.77 (0.44–1.36)	0.369	—	—
Medicaid	2.39 (0.64–8.98)	0.198	—	—
Uninsured	.	.	—	—
Other	1.49 (0.28–8.06)	0.642	—	—
Distance to treatment facility				
≤40 miles	1		—	—
>40 miles	1.23 (0.67–2.23)	0.503	—	—
Zip code education level				
≥21%	1		—	—
13%–20.9%	0.83 (0.35–1.97)	0.666	—	—
7%–12.9%	0.80 (0.35–1.84)	0.598	—	—
<7%	1.58 (0.66–3.82)	0.307	—	—
Zip code income level				
<38,000	1		—	—
38,000–47,999	0.72 (0.30–1.72)	0.460	—	—
48,000–62,999	0.95 (0.42–2.15)	0.896	—	—
≥63,000	1.27 (0.55–2.91)	0.572	—	—
Charlson/Deyo score				
0	1		—	—
1	0.64 (0.30–1.37)	0.256	—	—
2	0.43 (0.09–1.97)	0.274	—	—
3	1.71 (0.17–16.74)	0.646	—	—
Primary site				
Extremity	1		1	
Head and neck	0.54 (0.16–1.80)	0.314	0.45 (0.13–1.55)	0.205
Thorax	0.27 (0.11–0.67)	0.005	0.21 (0.08–0.55)	0.001
Abdomen/pelvis	0.26 (0.13–0.52)	<0.001	0.22 (0.10–0.45)	<0.001
Other/NOS	0.38 (0.02–6.30)	0.503	0.20 (0.01–3.39)	0.266
Tumor size				
<5 cm	1		—	—
5.1–10 cm	1.38 (0.69–2.79)	0.366	—	—
10.1–15 cm	1.45 (0.63–3.34)	0.385	—	—
>15 cm	0.72 (0.30–1.74)	0.470	—	—
Grade				
II	1		—	—
III	1.37 (0.40–4.63)	0.614	—	—
Receipt of chemotherapy				
No	1		—	—
Yes	1.32 (0.76–2.27)	0.321	—	—
Year of diagnosis				
2004–2007	1		—	—
2008–2011	0.61 (0.31–1.20)	0.151	—	—
2012–2015	1.09 (0.56–2.11)	0.806	—	—

## Data Availability

The data used to support the findings of this study are restricted by the National Cancer Database. Data are available from the NCDB for researchers who meet the criteria for access to the data as detailed at https://www.facs.org/quality-programs/cancer/ncdb/puf.
